# The Cancer Research UK Stratified Medicine Programme as a model for delivering personalised cancer care

**DOI:** 10.1038/s41416-022-02107-8

**Published:** 2023-01-04

**Authors:** Maria Antonietta Cerone, Tara C. Mills, Rowena Sharpe, David McBride, Moira MacDonald, Suzanne MacMahon, Hood Mugalaasi, Pauline Rehal, Alessandro Rettino, Helen Roberts, Mark Ross, Donald Edward White, John Peden, Janette Rawlinson, Steffan N. Ho, Simon Hollingsworth, Sanjay Popat, Gary Middleton, Peter Johnson, Charles Swanton, the SMP2 consortium, Somai Man, Rachel Butler, Rhian White, Sian Morgan, Sian Wood, Lisa Thompson, Hedley Carr, Sumi Subramaniam, Cian McGuire, Helen Pitman, Isabella Chen, Kirsty Tunna, Sahar Rehman, Catrin Middleton, Abdullah Alvi

**Affiliations:** 1 Cancer Research Horizons, CRUK, The Francis Crick Institute, London, UK; 2 Cancer Research UK, London, UK; 3 Cancer Research UK Clinical Trials Unit, University of Birmingham, Birmingham, UK; 4 Illumina Cambridge, Great Abington, Cambridge, UK; 5 All Wales Medical Genomics Service, University Hospital of Wales, Cardiff, UK; 6 The Centre for Molecular Pathology, The Royal Marsden, Sutton, UK; 7 West Midlands Regional Genetics Laboratory, Birmingham Women’s and Children’s NHS Foundation Trust, Birmingham, UK; 8 Genpax AG, Cambridge, UK; 9 Independent patient representative, Sandwell, West Midlands UK; 10 Pfizer, Global Product Development Oncology, San Diego, CA USA; 11 AstraZeneca, Late Development Oncology, Academy House, Cambridge, UK; 12 The Royal Marsden Hospital NHS Foundation Trust, London, UK; 13 Institute of Immunology & Immunotherapy, University of Birmingham, Birmingham, UK; 14 School of Cancer Sciences, University of Southampton, Southampton, UK; 15 The Francis Crick Institute, Midland Road, London, UK; 16 Wellcome Trust, London, UK; 17 Eli Lilly, London, UK; 18 British Heart Foundation, Bromley, UK; 19 Silence Therapeutics, London, UK; 20 Parexel, London, UK; 21 Ahead Care and Support, London, UK

**Keywords:** Biomarkers, Cancer genomics

## Abstract

Genomic screening is routinely used to guide the treatment of cancer patients in many countries. However, several multi-layered factors make this effort difficult to deliver within a clinically relevant timeframe. Here we share the learnings from the CRUK-funded Stratified Medicine Programme for advanced NSCLC patients, which could be useful to better plan future studies.

## Background

The use of genomic screening to guide the treatment of cancer patients is becoming routine. However, its implementation within complex healthcare systems is not without challenges.

Here we share the learnings from the CRUK Stratified Medicine Programme 2 (SMP2), a UK-wide genomic screening programme. Funded by Cancer Research UK (CRUK), the National Health Services (NHS) in the 4 UK Nations, AstraZeneca and Pfizer, SMP2 offered genomic screening to patients with advanced non-small cell lung cancer (NSCLC) for enrolment into the National Lung Matrix Trial (NLMT) [1, 2].

## What SMP2 delivered

**Fig. 1:**
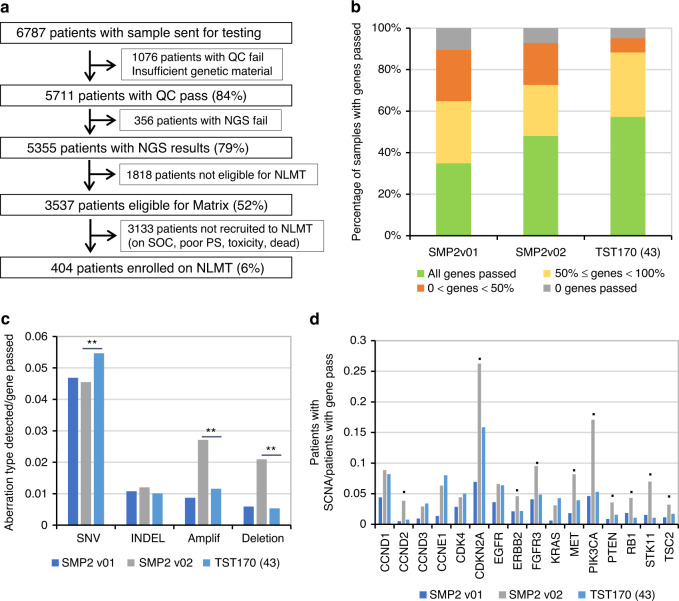
SMP2 at a glance. **a** SMP2 consort graph showing absolute numbers and percentage of tested patients. Patients not recruited to NLMT included patients on standard of care (SOC) treatment or dead at the time of closure, patients who could not be recruited due to poor performance status (PS) and patients who stopped treatment due to toxicity. **b** Comparison of NGS success rates for the 3 panels. The percentages of samples where the indicated fraction of genes was successfully sequenced are shown. All differences between panels are significant (*p* < 0.01). **c** Types of gene aberrations detected. The fractions of genes successfully sequenced in which the indicated aberration was detected over all genes passed are shown. **p < 0.01, Fisher’s exact test. **d** SCNA detection by panel. The fractions of patients where an amplification or deletion in the indicated genes was detected over the number of patients where the gene was successfully sequenced are shown. Only genes for which at least 50 SCNA events have been observed in the SMP2 cohort are included. ● = **; **p < 0.01, Fisher’s exact test.

SMP2 was the first study of its kind to be set up within the NHS at a time when next-generation sequencing (NGS) was just becoming clinically available [1–5]. The programme was successful in demonstrating the feasibility of delivering genomic testing at scale, with 79% of all patients tested having a genomic result (Fig. 1). This success was due to the collaborative effort between different stakeholders who worked together throughout the lifespan of the project to identify processes hindering it and implement changes to maximise screening success.

Indeed, between January 2015 and August 2021, over 10,000 patients were consented to SMP2 using a network of >50 hospitals spread throughout the country across diverse socio-economic backgrounds. Of these 6787 patients had a sample sent for testing while undergoing first-line standard-of-care (SOC) treatment. The overall turn-around-time (TAT) from patient consent to release of the genomic results was closely monitored and optimised to ensure that a molecular report would be available when patients relapsed on SOC and could be considered for NLMT enrolment [2] (median = 121 days). Different local processes at sites and poor sample quality account for most of the variability in the time needed from consent to sample sent for testing (median = 28 days, IQR = 27 days, 75% samples sent within 55 days), whereas the time required for testing at the molecular laboratories was stable (median = 19 days, IQR = 11 days, 75% reported within 26 days).

The ambition for SMP2 was to have a single assay capable of detecting all types of aberrations required for NLMT eligibility [2]. Therefore, a bespoke NGS panel (SMP2v01 panel) was designed by Illumina, covering the 28 genes proposed by the pharma partners [2]. The assay required sequencing of tumours and matched normal blood samples and could detect SNVs and indels at ≥10% frequency and SCNAs in samples with >60% tumour content.

To minimise the burden on patients, residual FFPE diagnostic samples were used for testing. Because of this, 20% of patients considered for SMP2 could not be tested due to insufficient tissue, and initially, 34% of samples could not be sequenced due to insufficient genomic material (QC fail). To reduce the QC fails and ensure that we could confirm the wild-type status of some NLMT genes, we set stringent thresholds for tumour content, read depth and coverage (20% tumour content, 500 reads across minimum 85% exons).

These changes dramatically reduced the fraction of samples that could not be sequenced from 34 to 15%, highlighting the importance of adequate sample processing. However, we still observed a significant NGS failure rate, with >10% of samples failing all 28 genes (sequencing coverage threshold not met) and an additional 25% failing up to 50% of the genes (Fig. 1b). Moreover, some genes failed significantly more frequently than others (*p* < 0.01), including *RB1*, which negatively impacted patients’ enrolment onto the NLMT as its wild-type status confirmation was an exclusion criterion for 1/3 of the cohorts [2].

In March 2017, the SMP2 panel was upgraded to SMP2v02 to optimise its performance and add target regions for a new NLMT arm. The upgrade included additional probes to improve coverage for genes with the highest failure rates and for calling SCNAs, fewer probes in highly repetitive intronic regions to reduce off-target effects and probes targeting SNPs to allow identification of blood-tumour mismatches. We also reduced the minimum allele frequency threshold from 10% to 5% and partly automated the analysis pipeline.

These changes resulted in a significant improvement in panel performance, with the overall gene failure rate (OFR) reducing from 36 to 28%. Also, the fraction of samples passing all genes markedly increased (35 to 48%) and the fractions of samples with >50% genes failed significantly decreased (Fig. 1b, all *p* < 0.01).

The need to allow new molecular arms for NLMT triggered a further panel upgrade in November 2019. We selected the Illumina TruSight Tumor 170 assay (TST170) as it queried DNA and RNA from the same sample, enabling the detection of gene fusions, reducing the need for FISH and the TAT. At the outset, although testing for all 170 genes, the molecular laboratories reported only 43 genes. The implementation of the TST170 panel required a significant change in sample preparation to enable both DNA and RNA workflows and did not require sequencing of the matched blood sample.

The new panel performed well with a further reduction of the OFR to 15%, an increase in the fraction of samples that passed all genes (48 to 57%) and a decrease of samples that failed >50% of the genes (27 to 12%, Fig. 1b, all *p* < 0.01). Overall, 88% of samples tested passed at least 50% of the genes compared to 73% on SMP2v02 panel and 65% on SMP2v01 panel (*p* < 0.01). This improvement is even more significant, considering that the number of genes reported on the new panel had almost doubled.

While the TST170 panel showed improvement in SNV calling (*p* < 0.01; Fig. 1c), it did not perform as well as the SMP2v02 panel for SCNAs in some genes frequently amplified or deleted in NSCLC (all *p* < 0.01; Fig. 1d); however, we accepted this limitation as the benefit of including more targets outweighed the potential loss in sensitivity.

## Conclusions

In summary, SMP2 demonstrated that routine genomic testing for NSCLC patients could be delivered at scale in a clinically relevant timeframe within a national health system. This has been achieved through an extensive infrastructure spread throughout the country to ensure access for all patients.

Our observations on processes for technology implementation, sample quality, logistics and reporting offer a useful resource to other groups setting up similar approaches [6].

From a technological point of view, our results highlight the importance of successive iterations of the screening pipeline to improve NGS success rate based on both biological and technological advances. Over the course of SMP2 we achieved this by taking a flexible approach to the type of panel and analysis used and by working collaboratively to implement changes successfully.

The main principle driving the success of SMP2 was putting the patients at the core of the research, and by facilitating access to the latest molecular diagnostics, patients could benefit from access to more personalised cancer treatments.

## Supplementary information

Full list of SMP2 Consortium
