# Correction to: The Cancer Research UK Stratified Medicine Programme as a model for delivering personalised cancer care

**DOI:** 10.1038/s41416-023-02160-x

**Published:** 2023-01-25

**Authors:** Maria Antonietta Cerone, Tara C. Mills, Rowena Sharpe, David McBride, Moira MacDonald, Suzanne MacMahon, Hood Mugalaasi, Pauline Rehal, Alessandro Rettino, Helen Roberts, Mark Ross, Donald Edward White, John Peden, Janette Rawlinson, Steffan N. Ho, Simon Hollingsworth, Sanjay Popat, Gary Middleton, Peter Johnson, Charles Swanton, the SMP2 consortium, Somai Man, Rachel Butler, Rhian White, Sian Morgan, Sian Wood, Lisa Thompson, Hedley Carr, Sumi Subramaniam, Cian McGuire, Helen Pitman, Isabella Chen, Kirsty Tunna, Sahar Rehman, Catrin Middleton, Abdullah Alvi

**Affiliations:** 1 Cancer Research Horizons, CRUK, The Francis Crick Institute, London, UK; 2 Cancer Research UK, London, UK; 3 Cancer Research UK Clinical Trials Unit, University of Birmingham, Birmingham, UK; 4 Illumina Cambridge, Great Abington, Cambridge, UK; 5 All Wales Medical Genomics Service, University Hospital of Wales, Cardiff, UK; 6 The Centre for Molecular Pathology, The Royal Marsden, Sutton, UK; 7 West Midlands Regional Genetics Laboratory, Birmingham Women’s and Children’s NHS Foundation Trust, Birmingham, UK; 8 Genpax AG, Cambridge, UK; 9 Independent patient representative, Sandwell, West Midlands UK; 10 Pfizer, Global Product Development Oncology, San Diego, CA USA; 11 AstraZeneca, Late Development Oncology, Academy House, Cambridge, UK; 12 The Royal Marsden Hospital NHS Foundation Trust, London, UK; 13 Institute of Immunology & Immunotherapy, University of Birmingham, Birmingham, UK; 14 School of Cancer Sciences, University of Southampton, Southampton, UK; 15 The Francis Crick Institute, Midland Road, London, UK; 16 Wellcome Trust, London, UK; 17 Eli Lilly, London, UK; 18 British Heart Foundation, Bromley, UK; 19 Silence Therapeutics, London, UK; 20 Parexel, London, UK; 21 Ahead Care and Support, London, UK

**Keywords:** Biomarkers, Cancer genomics

Correction to: *British Journal of Cancer* 10.1038/s41416-022-02107-8, published online 04 January 2023

The original version of this article contained an error in two author names. The correct names are Steffan N Ho and Janette Rawlinson. The authors apologize for the errors. The original article has been corrected.

