# Supplementary material for: The Cancer Research UK Stratified Medicine Programme as a model for delivering personalised cancer care
Source: Br J Cancer. 2023 Jan 4;128(2):161–4. doi: 10.1038/s41416-022-02107-8 (PMC9902467; doi:10.1038/s41416-022-02107-8)
Supplement: Supplementary file 1 — Full list of SMP2 Consortium [file 41416_2022_2107_MOESM1_ESM.docx]

**Full list of SMP2 Consortium**

Maria Antonietta Cerone^1^, Noor Afshan^2^, Pablo Olmos Aguirre^3^, Richard Adams^4^, Parry-Jones Alison^4^, Karen Allen^5^, Abdullah Alvi^6,37^, Stephanie Argue^7^, Ivana Armogida^3^, R Asher^8^, Liz Ashford^9^, Gareth Ayre^10^, Marina Baccarini^11^, Zoe Backholer^3^, Hayley Bair^2^, Simon Ball^11^, Caitlin Barry^12^, J. Bates^9^, Emma Bates^8^, Matt Baxter^10^, Mike Bayne^13^, James Beasley^2^, Selina Begum^13^, Janie Bell^14^, Stefano Berri^3^, Angela Berry^5^, Jaishree Bhosle^15^, Lesley Bishop^13^, Fiona Blackhall^16^, Fiona Black^12^, Lucy Blackwell^12^, Richard Booton^16^, Ruth Boyd^5^, Alison Brewster^4^, Alison Bridgewood^12^, Rachel Browell^12^, Nick Brown^9^, Gary Brown^7^, Lisa-J Brown-Schofield^12^, Jonathan Bury^8^, Rachel Butler^17^, Peter Campbell^3^, Paul Cane^27^, Hedley Carr^18^, Mathew Carter^16^, David Gonzalez de Castro^17^, Judith Cave^13^, Nicola Charles^8^, Anshuman Chaturvedi^16^, Isabella Chen^6,31^, Heather Cheyne^8^, Dominika Chwialkowska^12^, Charles Comins^10^, John Conibear^11^, Rory Convery^5^, A Cooney^8^, Michael Cornwell^19^, Nicholas Coupe^20^, Niamh Cronin^11^, Wanda Cui^15^, Adam Dangoor^10^, Sarah Danson^8^, Tathagata Das^8^, Elizabeth Davey^29^, Michael Davidson^15^, Tom Newton Davies^15^, Alison Davison^22^, Kay Dawson^24^, Veronica Delogu^15^, Jo Deubert^15^, Craig Dick^23^, Rachel Doak^2^, James Dobbyn^15^, Ronan Donnelly^5^, Nicole Dorey^29^, Stacey Duffy^12^, Faith Dzumbunu^11^, Dawn Edwards^29^, Douglas Elkin^21^, Dean A Fennell^21^, Paul Fenton^13^, Victoria Ferguson^12^, Barbara Figueiredo^15^, Patricia Fisher^8^, Vicky Ford^29^, Martin Forster^7^, Mel Foster^13^, Grace Fryer^12^, Tom Geldart^13^, Qamar Ghafoor^14^, Angela Green^8^, Kristy Lee Greene^5^, Michelle Greenhalgh^16^, Alastair Greystoke^12^, Richard Griffith^21^, Emma Griffiths^8^, Mike Griffiths^2^, Rebecca Grogan^21^, Abdulqader Hadi W^10^, Graham Halford^2^, Elizabeth Happle^13^, Sara Harrison^21^, Matthew Hatton^8^, Lisa Head^9^, Amber Heeley^16^, Libby Hennessy^15^, Hayley Hewer^28^, Linda Hogarth^12^, Abigail Hollingdale^19^, Papawadeen Ingram^13^, Charlotte Jacobs^12^, Amanda Jackson^4^, Jackie James^5^, Albert Jimenez-Tomas^10^, Rosemary Johnson^15^, Benjamin Johnson^13^, Christopher Jones^12^, Srivani Kandasamy^27^, Andrea Kay^12^, K. Kerr^8^, Emma King^12^, Pek Koh^14^, Muthu Kumar^28^, Rajiv Kumar^15^, Matthew Lane^12^, Stephen Lang^10^, Mary Jane Lauigan^15^, Theresa Lawless^29^, Caroline Lee^8^, Linda Leith^8^, Cathryn Leng^8^, Claire Lewis^5^, Steven Liggett^12^, Colin Lindsay^16^, Louise Li^12^, R Lloyd^8^, Margarida Lopes^3^, T. Lowe^12^, Abigail MacArthur^4^, Venkat Mahadevan^19^, David Maldonado-Perez^20^, Somai Man^2^, Alexander Martin^19^, Helen Martin^13^, Sanjanah Masinghe^12^, Malcom Mason^4^, Manisha Maurya^17^, Nicole Maziere^21^, Beverley Mcclelland^12^, Sara McCusker^5^, Eve Mcgarry^22^, Adam Mcgeoch^19^, Cian McGuire^6,31^, Austin McInnes^23^, Ian Mcneish^24^, Rachel Merry^7^, Catrin Middleton^6,37^, Jenny Miller^21^, Charlotte Miller^8^, Charlotte Milner-Watts^15^, Anna Minchom^15^, Jennifer Moor^11^, J. Moore^12^, Fiona Morgan^4^, Helen Morgan^15^, Sian Morgan^25^, Dion Morton^14^, Georgia Moule^19^, Lois Mulholland^5^, Paula Mulvenna^12^, Mamoona Munir^2^, Tracey Murray^19^, Daniel Naeh^4^, Kerry Nicholls^5^, Sarah Nicholson^21^, Andrew Nicholson^15^, Pamela Niem^7^, Glenn Noel-Storr^15^, Luke Nolan^13^, Catherine Norman^11^, Mary O’Brien^15^, Hazel O’Sullivan^15^, Maeve O’Driscoll^24^, Clare Orange^23^, Waheeda Owadally^10^, Sangeeta Paisey^13^, Krishna Patel^7^, Dan Patterson^19^, Mary Perrin^9^, Maria Piga^15^, Helen Pitman^6,33^, Danuta Plumer^15^, Lynda Poole^12^, Kathryn Potts^12^, Manish Powari^29^, Vasanthi Prathapan^15^, Gillian Price^8^, Kathryn Priest^15^, Jeanette Raine^12^, Doris Rassl^19^, Sahar Rehman^6,34^, Louise Renouf^17^, Shirley Richardson^8^, Bianca Rock^15^, Sue Roffe^13^, Jane Rogan^16^, Philip Russell^16^, Imran Sadat^23^, Derya Sahin^15^, Katrin Sainudeen^15^, Janine Salter^15^, Joshua Savage^30^, Lydia Scarlett^13^, k. Shackcloth^16^, Riyaz Shah^22^, Paul Shaw^4^, Emily Shaw^13^, L Sheazadi^8^, Suzanne Sheppard^13^, Magdalena Shields^2^, Timothy Simmons^12^, Matthew Smith^14^, Jackie Smith^13^, Matt Smith^2^, Azmina Sodha-Ramdeen^21^, James Spicer^27^, Katherina Sreter^15^, Natalie Stacey^4^, Kimberly Stamp^12^, Jane Steele^14^, Nicola Steele^23^, Coakley Steph^4^, Amanda Stone^19^, Sophia Strong-Sheldrake^13^, Sumi Subramanian^6,35^, Yvonne Summers^16^, Megan Tait^23^, Denis Talbot^20^, Phillipe Taniere^14^, Paul Tate^21^, Fiona Taylor^8^, Joyce Thompson^14^, Y Thompson^8^, Catherine Thompson^13^, Lisa Thompson^17^, Lucy Thorogood^28^, Nadza Tokaca^15^, Peter Tovey^28^, Liz Toy^29^, Helen Trower^8^, Kirsty Tunna^6,36^, Emma Turay^15^, Helen Turnbull^12^, Finn Tysoe^20^, Deniz Ucanok^2^, Eldo Verghese^9^, Alexandra Vick^15^, S. Wadd^12^, Fiona Wakinshaw^12^, C. Walker^12^, J.C. Walters^13^, Anya Wandowski^13^, Andrea Watson^12^, Paul Westwood^23^, Laura White^15^, Rhian White^25^, Karen Wild^4^, Beverley Wilkinson^12^, Deborah Willcox^22^, Christine Willshire^23^, Rob Wilson^4^, G.D. Wilson^8^, Lisa Wood^19^, Sian Wood^25^, Andrea Worsdale^12^, Andrew Wotherspoon^17^, Ashlene Wright^6^, Kent Yip^19^, Agnieszka Yongue^15^, Robin Young^8^, Nadia Yousaf^15^, Ishtiaq Zubairi^8^

^1^Cancer Research Horizons, CRUK, The Francis Crick Institute, London, UK. ^2^Birmingham Women’s and Children’s NHS Foundation Trust, West Midlands Regional Genetics Laboratory, Birmingham, UK. ^3^Illumina Cambridge, Great Abington, Cambridge, UK. ^4^Velindre University NHS Trust, Cardiff, UK. ^5^Queen’s University Belfast, Belfast, NI, UK. ^6^Cancer Research UK, London, UK. ^7^University College Hospital, UCL, London, UK. ^8^Weston Park Cancer Centre, Sheffield, UK. ^9^Leeds Teaching Hospital NHS Trust, Leeds, UK. ^10^University Hospitals Bristol NHS Foundation Trust. ^11^St Bartholomew’s Hospital, London, UK. ^12^Newcastle Northern Centre for Cancer Care, Newcastle University, Newcastle, UK. ^13^University Hospital of Southampton NHS Foundation Trust, Southampton, UK. ^14^University Hospital Birmingham, Birmingham, UK. ^15^The Royal Marsden Hospital NHS Foundation Trust, London, UK. ^16^The Christie NHS Foundation Trust, Manchester, UK. ^17^The Centre for Molecular Pathology, The Royal Marsden, Sutton, UK. ^18^AstraZeneca, Academy House, Cambridge, UK. ^19^Royal Papworth Hospital NHS Foundation Trust, Cambridge, UK. ^20^Oxford University Hospital NHS Foundation Trust, Oxford, UK. ^21^Clatterbridge Cancer Centre NHS Foundation Trust, Clatterbridge, UK. ^22^Maidstone and Tunbridge Wells NHS Trust, Tunbridge Wells, UK. ^23^Queen Elisabeth University Hospital, Glasgow, UK. ^24^Imperial College London, London, UK. ^25^All Wales Medical Genomics Service, University Hospital of Wales, Cardiff, UK. ^26^Leicester Cancer Research Centre, Leicester, UK. ^27^Guy’s and St Thomas’ NHS Foundation Trust, King’s College London, London, UK. ^28^East Suffolk and North Essex NHS Foundation Trust, Colchester, UK. ^29^Royal Devon and Exeter NHS Foundation Trust, Exeter, UK. ^30^Cancer Research UK Clinical Trials Unit, University of Birmingham, Birmingham, UK. ^31^Silence Therapeutics plc, London, UK. ^32^Eli Lilly, London, UK. ^33^British Heart Foundation, Bromley, UK. ^34^Ahead Care and Support, London, UK. ^35^Wellcome Trust, London, UK. ^36^Parexel, London, UK. ^37^Unaffiliated author.
